# Effect of increased enteral protein intake on plasma and urinary urea concentrations in preterm infants born at < 32 weeks gestation and < 1500 g birth weight enrolled in a randomized controlled trial – a secondary analysis

**DOI:** 10.1186/s12887-018-1136-5

**Published:** 2018-05-08

**Authors:** Michaela Mathes, Christoph Maas, Christine Bleeker, Julia Vek, Wolfgang Bernhard, Andreas Peter, Christian F. Poets, Axel R. Franz

**Affiliations:** 1 Department of Neonatology, University Children’s Hospital, Tübingen University Hospital, Calwerstr. 7, Tübingen, Germany; 2 Center for Pediatric Clinical Studies, University Children’s Hospital, Tübingen University Hospital, Tübingen, Germany; 3 Department of Internal Medicine IV, Division of Endocrinology, Diabetology, Vascular Medicine, Nephrology and Clinical Chemistry and Pathobiochemistry, University of Tuebingen, Tübingen, Germany; 4 Institute of Diabetes Research and Metabolic Diseases (IDM) of the Helmholtz Center Munich at the University of Tuebingen, Tübingen, Germany; 5 German Center for Diabetes Research (DZD), Muenchen-Neuherberg, Germany

**Keywords:** Infant, premature, Very low birth weight infant, Infant, newborn, Enteral feeding, Nutrition, Protein supply, Milk, human, Supplementation, Targeted fortification, Urea concentration

## Abstract

**Background:**

Feeding breast milk is associated with reduced morbidity and mortality, as well as improved neurodevelopmental outcome but does not meet the high nutritional requirements of preterm infants. Both plasma and urinary urea concentrations represent amino acid oxidation and low concentrations may indicate insufficient protein supply.

This study assesses the effect of different levels of enteral protein on plasma and urinary urea concentrations and determines if the urinary urea-creatinine ratio provides reliable information about the protein status of preterm infants.

**Methods:**

Sixty preterm infants (birthweight < 1500 g; gestational age < 32 weeks) were enrolled in a randomized controlled trial and assigned to either a lower-protein group (median protein intake 3.7 g/kg/d) or a higher-protein group (median protein intake 4,3 g/kg/d). Half the patients in the higher-protein group received standardized supplementation with a supplement adding 1.8 g protein/100 ml milk, the other half received individual supplementation depending on the respective mother’s milk macronutrient content. Plasma urea concentration was determined in two scheduled blood samples (BS1; BS2); urinary urea and creatinine concentrations in weekly spot urine samples.

**Results:**

The higher-protein group showed higher plasma urea concentrations in both BS1 and BS2 and a higher urinary urea-creatinine-ratio in week 3 and 5–7 compared to the lower-protein group. In addition, a highly positive correlation between plasma urea concentrations and the urinary urea-creatinine-ratio (*p* < 0.0001) and between actual protein intake and plasma urea concentrations and the urinary urea-creatinine-ratio (both *p* < 0.0001) was shown.

**Conclusions:**

The urinary urea-creatinine-ratio, just like plasma urea concentrations, may help to estimate actual protein supply, absorption and oxidation in preterm infants and, additionally, can be determined non-invasively. Further investigations are needed to determine reliable cut-off values of urinary urea concentrations to ensure appropriate protein intake.

**Trial registration:**

Clinicaltrials.gov; NCT01773902 registered 15 January 2013, retrospectively registered.

## Background

Feeding human milk has a lot of beneficial effects on preterm and term newborn infants. It contains immunoglobulins and cytokines and therefore offers protection against infections [1–3]. Besides, the ingestion of maternal milk reduces the incidence of necrotizing enterocolitis [4, 5], retinopathy of prematurity [5] and diabetes type 1 and 2, as well as obesity in later life [6, 7]. Furthermore, it is associated with improved neurocognitive and psychomotor development [8]. Therefore, mother’s milk is considered the optimal nutritional source for preterm infants [9]. However, human milk does not meet the high nutrient needs of preterm infants, hence requires supplementation with multicomponent fortifiers [10]. Nevertheless, many preterm infants show early postnatal growth retardation despite having been fed fortified human milk [11].

In general, an average composition of breast milk is assumed and fortifiers are added in a standardized manner, but this assumption is rarely appropriate [12], because human milk is a biological product with considerable intra- and inter-individual variation of its macro- and micronutrient content, particularly protein [13].

To ensure an optimal supplementation with protein in the daily neonatal intensive care unit (NICU) routine, it is necessary to establish a feasible method to assess the individual protein supply and, ideally, protein availability for growth in very preterm infants.

**Fig. 1:**
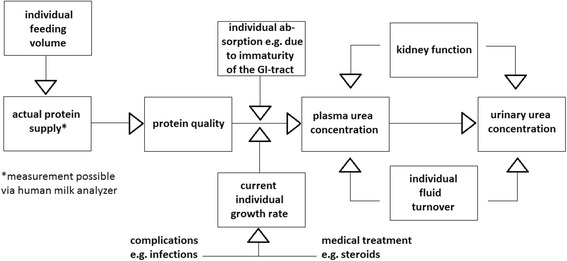
Concept of the interaction of actual protein intake and growth on plasma and urinary urea concentrations

Frequent measurements of the individual breast milk macronutrient content using a milk analyzer (mid-infrared spectroscopy) is an easy and valid method to assess macronutrient supply [14]. Nevertheless, due to the immaturity of the gastrointestinal system of a preterm infant and the individual intestinal microbiome the calculation of actual macronutrient supply might not reflect the effectively absorbed amount of protein, fat and energy (‘actual intake’). Our concept of the interaction of actual protein intake and growth on plasma (PUC) and urinary urea concentrations (UUC) is illustrated in Fig. 1.

The PUC represents amino acid oxidation and therefore offers information about availability of protein after absorption and incorporation for growth, provided that fluid intake, kidney function and quality of the nutritional protein remain unchanged. However, associated blood samples are painful, contribute to blood loss and carry the risk for additional complications. In contrast, urine can be collected non-invasively and painlessly, and is frequently collected to guide calcium and phosphate supplementation in preterm infants [15]. Because the UUC may be confounded by fluid homeostasis, normalization of urinary urea for the creatinine excretion as the urinary-urea-creatinine-ratio (UUCR) may be appropriate.

The aim of this study was to determine 1) the impact of increased enteral protein intake on PUC and UUCR in very preterm infants and 2) if the UUCR represent PUCs and actual enteral protein supply in this population.

## Methods

Detailed information on the underlying randomized controlled trial has been previously reported [16]; in short**:**

From October 2012 to October 2014, this partially blinded randomized controlled trial was conducted at the department of neonatology at Tübingen university children’s hospital, Germany. The Institutional Review Board approved the protocol and written informed parental consent was obtained. The trial was registered with Clinicaltrials.gov as NCT01773902.

The primary objective of the randomized controlled trial was to evaluate the effects of different target levels of enteral protein supplementation on growth in predominantly human milk fed very preterm infants [16], where no significant difference regarding the growth velocity (g/kg/d) from birth to end of intervention was shown. Both groups achieved near-fetal growth-rates. Secondary objectives of this trial were analysis of UUCR and PUC.

### Participants

Sixty preterm infants born at our hospital within the above time frame, and with gestational age < 32 weeks at birth and weight < 1500 g were included. Main exclusion criteria were major congenital or chromosomal abnormalities.

### Intervention

Infants were randomly assigned in proportions of 2/1/1 to one of three parallel treatment groups (after reaching 100 ml/kg enteral nutrition per day and informed consent was given) including:

1) a lower-protein group (LPG) (standardized fortification adding 5 g/100 ml of the commercially available multicomponent fortifier FM 85**®** (Nestlé Nutrition, Frankfurt, Germany; the standard fortifier in our unit, ingredients shown in Table 1) resulting in supplementation of 1 g protein/100 ml);

**Table 1:** Ingredients of the multicomponent fortifiers

	5 g BEBA FM 85	5 g Study fortifier 10.01.DE.INF
	74 kJ / 18 kcal	90 kJ / 22 kcal
Protein	1,0 g	1,8 g
Total carbohydrates (including:)	3,3 g	1,8 g
lactose	0 g	0 g
maltodextrin	3,2 g	1,8 g
Other carbohydrates	0,1 g	0 g
Total lipids (incuding:)	0,02 g	0,87 g
docosahexaenic acid (DHA)		0,0075 g
arachidonic acid (AA)		0,0006 g
Sodium	20 mg	33 mg
Potassium	42 mg	83 mg
Chloride	17 mg	29 mg
Calcium	75 mg	94 mg
Phosphor	45 mg	56 mg
Magnesium	2 mg	5 mg
Iron	1,3 mg	0,94 mg
Copper	0,04 mg	0 mg
Zinc	0,8 mg	0 mg
Iodine	15 μg	19 μg
Selenium	1,5 μg	4,25 μg
Vitamin A	0,15 mg	0,5 mg
Vitamin D	2,5 μg	5 μg
Vitamin E	2,0 mg	5 mg
Vitamin K	4,0 μg	10 μg
Vitamin C	10 mg	25 mg
Vitamin B1	0,05 mg	0,19 mg
Vitamin B2	0,10 mg	0,25 mg
Vitamin B6	0,05 mg	0,16 mg
Folic Acid	40 μg	50 μg
Vitamin B5	0,4 mg	0,88 mg
Vitamin B12	0,1 μg	0,25 μg
Biotin	3 μg	4 μg

2) a higher-protein group (HPG) which comprised subgroups:

2a) with standardized higher protein supplementation using 5 g/100 ml of an investigational multicomponent fortifier. The study fortifier administered to group 2a) contained 1.8 g protein/5 g fortifier (10.01.DE.INF, Nestlé Nutrition, Frankfurt, Germany, ingredients shown in Table 1).

2b) individually adjusted fortification based on the individual human milk macronutrient content on top of standardized fortification as in group 1.

In all three study groups, multicomponent fortifier was added at a fixed dose of 2.5 g/100 ml breast milk at enteral intakes between 100 and 149 ml/kg/d and at 5 g/100 ml breast milk once ≥150 ml/kg/d of enteral feeds had been reached and always thereafter.

In infants randomized to individual fortification (group 2b), the protein dosage was adjusted according to breast milk content aiming for 4.5 g/kg/d of enteral protein if the weight was < 1500 g or 4.0 g/kg/d of enteral protein if the weight was > 1500 g according to recommendations of the European Society of Paediatric Gastroenterology, Hepatology and Nutrition [17]. Moreover, fat was supplemented to ensure a cumulative fat intake > 4.8 g/kg/d. Aptamil Eiweiß+®, (Milupa, Friedrichsdorf, Germany) was used as an additional protein source in individually supplemented infants and a generic medium-chain triglyceride oil (Oleum neutrale) was used as an additional source of enteral fat. In the following, group 2a (mean protein intake 4,38 g/kg/d) and 2b (mean protein intake 4,12 g/kg/d) were combined (HPG; mean protein intake 4,30 g/kg/d) for further investigations.

### Measurement of serum and urine urea

According to the study protocol, venous blood samples to measure PUC were scheduled on day 14 (±2) and day 28 (±4) after randomization, but had to be re-scheduled until clinical indication arose to avoid study-driven needle-sticks, which resulted in an earlier collection of BS 1. BS 1 was taken on day 9 (7–10) after randomization, BS 2 on day 27 (24–32). Since UUCs were measured weekly, the closest urine sample (BS max. + / - 3 days) was matched to each blood sample. For BS1 sample size was 54; for BS2 sample size was 41 (available serum-urine-urea-pair).

To take the individual fluid turnover of the preterm infants into account UUCs are shown as urinary urea to urinary creatinine ratio (urea concentration (mg/dl) /creatinine concentration (mg/dl) of the same urine sample) (UUCR).

Every week the urinary urea and creatinine concentrations were measured from clinically indicated spot urine samples used for guidance of calcium and phosphorus supplementation as described in [15]. Urine was collected non-invasively with an absorbent fleece placed in the diaper.

### Biochemical analyses

Determination of PUC and UUC was performed on the ADVIA XPT clinical chemistry analyzer (Siemens Healtheneers, Eschborn, Germany). Internal and external quality controls were always within the allowed ranges. The inter assay coefficient of variation was < 2.5% for plasma and < 4% for urine.

### Actual daily protein supply

The median actual daily protein supply (based on mother’s milk protein content (measured twice a week with a human milk analyzer (Miris, Uppsala, Sweden; infrared spectroscopy; calibration was carried out once a day as recommended by the manufacturer using a check solution); formula’s protein content, protein content of the fortifier and parenteral amino acid supply) was calculated for the 5 days prior to the respective blood sample to ensure that variations due to clinical nutrition restriction/ lack of mother’s milk were taken into account.

Single samples of breast milk were used to measure macronutrient content as the mean of three individual measurements.

### Statistical analyses

Statistical analyses were performed using Microsoft Excel and the statistic software JMP. Data was tested with non-parametric Wilcoxon-Test and Spearman’s rank correlation coefficient (rho) was used for analysis of correlation, since not all data showed normal distribution. Results are shown as median and p25-p75.

## Results

There were no differences in birth weight, gestational age at birth, gender or length of hospital stay between the LPG and the HPG [16], no statistic differences regarding the comorbidities due to early preterm birth between the two groups were shown (Table 2). The HPG had a significant higher protein supply while no difference in median energy supply was shown considering the time from birth to end of intervention and no difference in weight gain was shown (Table 3).

**Table 2:** Comparison of the comorbidities of LPG and HPG

	LPG	HPG	*p*-value
NEC (≥ Bell stage 2A)	0(0)	0(0)	1
PDA therapy (indomethacin or ibuprofen)	4(13)	3(10)	1
BPD (physiological definition^a^)	0(0)	2(7)	0,49
ROP^b^/^c^	0(0)	1(3)	1
IVH^d^	3(10)	0(0)	0,24
PVL / intra-parenchymal bleeding	0(0) / 0(0)	0(0) / 0(0)	1 / 1
Corticosteroid administration inhaled / systemic^e^	0(0) / 1(3)	5(17) / 1(3)	0,05 / 1

^a^moderate or severe BPD indicated by need for positive pressure respiratory support or supplemental oxygen at a postmenstrual age of 36 weeks to maintain an SpO2 > 90%, verified by a room air test where indicated

^b^no ophthalmologic examination in 6 patients

^c^max. Stage of ROP in all participants: stage I

^d^max. Grade of IVH in all participants: grade I

^e^budesonide inhalation, systemic supply of hydrocortisone (no patient received dexamethasone)

**Table 3:** Patient details lower-protein group vs. higher-protein group, data shown as number n (%), respectively median (p25-p75), or *mean (±SD)

	Lower-protein group	Higher-protein group	*p*-value
Sex female	19(63)	14(47)	0,3
Gestational age in weeks	30,0(29,0–31,1)	29,7(27,9–31,0)	0,2
Birth weight in g	1215(1065–1393)	1193(984–1326)	0,61
Day of randomisation in days after birth	7(6–7)	7(6–8)	0,75
Length of hospital stay	52(42–65)	52(37–70)	0,6
Mean protein supply in g/kg/d (birth to end of intervention)	3,82(3,59–3,93)	4,30(4,11–4,43)	< 0,0001
Mean energy supply in kcal/kg/d (birth to end of intervention)	136(133–143)	137(135–147)	0,62
Weight gain in g/kg/d (birth to end of intervention, primary outcome of the underlying study)*	16,25 (±2,22)*	16,02 (±2,48)*	0,71

**Fig. 2:**
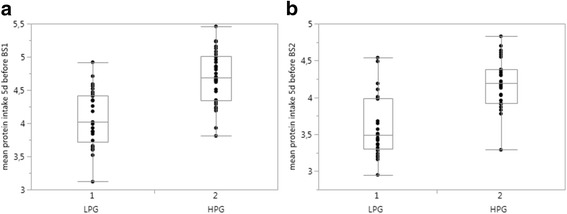
Protein intake 5 days before blood samples (BS) were taken **a** BS1: lower-protein group (4.02 (3.72–4.42)) vs. higher-protein group (4.69 (4.34–5.01)) *p* < 0.001; **b** BS2 - lower protein group (3.49 (3.30–3.99)) vs. higher-protein group (4.19 (3.92–4.38)) *p* < 0.0001

**Fig. 3:**
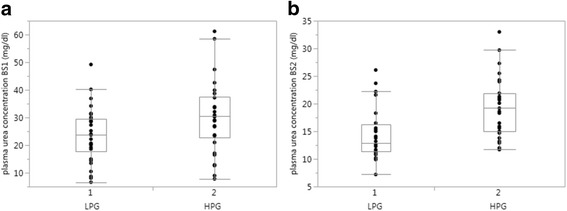
Plasma urea (PU) concentrations (mg/dl) – **a** Blood sample (BS) 1 lower-protein group 23.9 (17.7–29.6)) vs. higher-protein group 30.6 (22.8–37.6) *p* = 0.03; **b** BS2 lower-protein group 12.9 (11.4–16.3) vs. higher-protein group 19.2 (15.0–21.9) *p* = 0.0008

In comparison to the LPG, the HPG had also a significantly higher mean actual daily protein supply 5 days before blood sampling (Fig. 2); accordingly, in both samples higher PUC in the HPG were detected (Fig. 3).

The UUCR in the whole cohort ranged from 3.7 to 87.1 (median: 30.0) with a 2.5 Percentile of 9.3.

The UUCRs in both groups were similar in week 2 of life, but significantly higher in the HPG in week 3, as well as in weeks 5 through 7, with a trend to higher UUCRs in the HPG in week 4 (*p* = 0.08) (Table 4).

**Table 4:** Urinary urea-creatinine-ratio in the lower protein group versus high protein group in week two to seven after birth, showing significant difference in week 3 and from week 5 to week 7

Weeks	Lower-protein group urinary urea-creatinine-ratio	Higher-protein group urinary urea-creatinine-ratio	*p*-value
2	38,0 (24,1–52,7)	43,3 (30,2–49,5)	0.77
3	25,8 (16,9–41,1)	39,9 (33,5–51,0)	0.0019
4	26,2 (20,7–31,3)	30,9 (24,4–46,5)	0.086
5	21,0 (14,9–26,0)	30,7 (26,2–38,5)	0.0024
6	25,3 (17,4–29,6)	33,7 (25,1–42,2)	0.029
7	16,3 (12,1–30,8)	34,0 (27,8–52,0)	0.0083

**Fig. 4:**
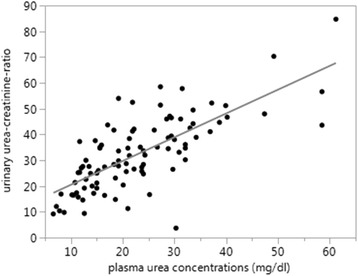
Plasma urea concentration in mg/dl and Urinary urea-creatinine-ratio (UUCR) show highly significant positive correlation (*p* < 0.0001), *ρ* = 0.72; y = 11.28407 + 0.9221688*x

UUCRs and PUCs showed a highly significant positive correlation when all samples were compared (*p* < 0.0001), with ρ = 0.72 (Fig. 4).

**Fig. 5:**
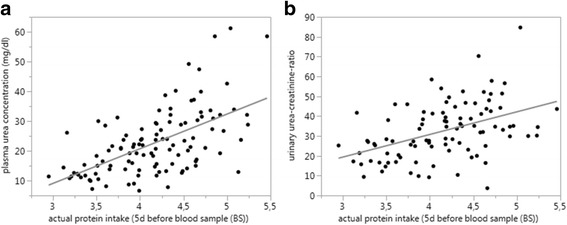
Actual protein intake (5d before blood sample (BS)) and **a** Plasma urea concentration (*p* < 0.0001; ρ = 0.59); y = − 25.8499 + 11.644884*x and **b** Urinary urea-creatinine ratio (UUCR) (*p* < 0.0001; *ρ* = 0.47) showed highly significant positive correlation; y = − 14.80586 + 11.401612*x

In addition, median actual daily protein intake in the last 5 days before BS was calculated and PUC as well as the matched UUCR samples showed a highly significant positive correlation (Fig. 5).

No correlation of median daily growth rate during 5 days prior to BS was observed with PUC (*p* = 0.7; *ρ* = − 0.03) and UCC (*p* = 0.56; ρ = − 0.04) in this generally well-thriving cohort (Difference of weight-SDS between end of intervention and birth were LPG: 0.26 (− 0.18–0.60) and HPG: 0.13 (− 0.11–0.60), respectively).

## Discussion

Inadequate postnatal enteral protein supply (and absorption, hence intake) may contribute to early postnatal growth failure of preterm infants [18], which may be associated with poor neurocognitive outcome in later life [19]. In cases of intrauterine growth retardation, reduced plasma amino acid concentrations were observed in cord blood samples during the third trimester [20–22].

At the contrary, an excess in protein supply may no longer improve growth [16, 23] and may theoretically also have adverse effects and should therefore be avoided.

Beyond defining the actual protein supply to a preterm infant, the immaturity of the gastrointestinal tract (as well as the individual microbiome) may impair individual absorption of administered protein. Therefore, further information is required to estimate whether the actual protein intake is adequate, i.e. to determine whether additional protein supply may help to optimize growth or is inadequate because the infant already oxidizes excessively administered protein.

High enteral protein intake results in higher blood urea nitrogen levels in preterm infants, which can be used as a guidance to estimate optimal protein supply in enterally fed preterm infants. In addition, an excess of protein leads to higher PUCs and therefore high PUCs might identify a surplus of protein [16, 23].

Our investigations show a highly significant correlation between PUCs and UUCRs and in addition a highly significant positive correlation between UUCRs and PUCs and actual protein intake in fairly stable fully enterally fed very preterm infants.

Measuring UUCs and its normalization for creatinine excretion as UUCRs, as indicator (surrogate parameter) of amino acid oxidation, would be an easy, non-invasive way to estimate oxidation of surplus protein (as result of enteral protein supply and absorption and growth) in preterm infants. Measuring UUCs is already used in older children and adults to evaluate protein turn-over and intake, e.g. in the context of renal failure. In the late 1960’s, a decrease in UUCs during periods of prolonged starvation was detected in adults [24], and therefore UUCR could become a marker for protein malnutrition in preterm infants, too. Polberger et al. showed in 1990 that low protein intakes result in low serum and urinary urea concentrations, and vice versa [25]. Despite a lack of good correlation between parenteral supply of protein and blood urea nitrogen levels in unstable preterm infants during the first days of life [26], Roggero et al. identified blood urea nitrogen as a potential marker of protein intake in low-birth-weight preterm infants on full enteral feeds [27]. The major advantage of assessing UUCRs is that urine is regularly disposed with the diapers; non-invasive collection of urine is painless and does not bear any risk for the infant. In our institution, urine collection is performed every week to assess calcium and phosphate metabolism in very low birth weight infants [15], so routine measurements of UUCR can be easily established. Considering that preterm infants continuously receive enteral nutrition every 2–3 h, spot urine sampling is considered to be sufficiently precise regarding the mineral homeostasis in a stable preterm infant [28] and may be sufficient to assess UUCRs as well.

Patients from this study with an actual protein supply of 4.0–4.5 g/kg/d during the 5 days prior to BS, standardized feeding regimens and near-optimal growth along their intrauterine birth trajectories [16], had UUCRs ranging from 3.7 to 84.7 (min; max); median: 36.1. This wide range indicates that variability of protein oxidation and urea elimination is high, even if protein supply is adequate, probably due to variability in protein absorption, growth rate, need for protein oxidation for gluconeogenesis and energy metabolism, as well as kidney function. Consequently, confounding factors as well as mediators and modifiers need to be considered when interpreting UUCRs (Fig. 1).

In this well thriving cohort (SDS-difference for weight between birth and end of intervention 0.18 (− 0.165–0.59)) [16] with adequate actual protein supply 4.0 (3.8–4.3) g/kg/d the 2.5 percentile of UUCR was 9.3. Lower UUCRs (< 9 mg/mg) may indicate a low protein availability. Hence in preterm infants with inadequate weight gain and UUCRs < 9 insufficient protein availability should be considered as cause for growth failure.

Nevertheless, further investigations addressing the before mentioned confounding factors and mediators in a larger population are needed to determine reliable cut-off values and to establish an algorithm based on UUCR and confounding factors to indicate adequacy of protein intake in clinical routine.

### Limitations and strengths of the study

In this randomized controlled study, the primary goal was to determine the effect of two levels of protein supply on weight gain. Nitrogen intake and balance weren’t measured directly and the study population lacks infants with clearly insufficient protein intake. At the contrary, actual protein intakes, weight gain and longitudinal growth, PUCs and UCCs (and respectively UUCRs) were assessed prospectively, and the whole cohort showed near-intrauterine weight gain throughout the study.

## Conclusion

In very preterm infants, determination of urinary urea/urinary creatinine ratios (UUCR) is a promising approach to estimate adequacy of protein supply non-invasively, since urinary urea/urinary creatinine ratios show highly positive correlation with plasma urea concentrations and actual protein supply.

## Abbreviations

BS: Blood sample
PUC: Plasma urea concentration
UUC: Urinary urea concentration
UUCR: Urinary urea-creatinine-ratio
LPG: Lower-protein group
HPG: Higher-protein group
Fig.: Figure
SDS: Standard deviation score
NEC: Necrotizing enterocolitis
PDA: Patent ductus arteriosus
BPD: Bronchopulmonary dysplasia
ROP: Retinopathy of prematurity
IVH: Intraventricular hemorrhage
